# Quality of Life in Patients with High-grade Non–muscle-invasive Bladder Cancer Undergoing Standard Versus Reduced Frequency of Bacillus Calmette-Guérin Instillations: The EAU-RF NIMBUS Trial

**DOI:** 10.1016/j.euros.2023.08.004

**Published:** 2023-09-12

**Authors:** Christine G.J.I. van Straten, Christien Caris, Marc-Oliver Grimm, Marc Colombel, Tim Muilwijk, Luis Martínez-Piñeiro, Marko M. Babjuk, Levent N. Türkeri, Joan Palou, Anup Patel, Anders S. Bjartell, Wim P.J. Witjes, Antoine G. van der Heijden, Lambertus A.L.M. Kiemeney

**Affiliations:** a Department of Urology, Radboud University Medical Center, Nijmegen, The Netherlands; b EAU Research Foundation, Arnhem, The Netherlands; c Department of Urology, Jena University Hospital, Jena, Germany; d Department of Urology, Hospital Edouard Herriot, Lyon, France; e Department of Urology, University Hospitals Leuven, Leuven, Belgium; f Department of Urology, Hospital Universitario La Paz, Madrid, Spain; g Department of Urology, Hospital Motol, 2nd Faculty of Medicine, Charles University, Prague, Czech Republic; h Department of Urology, Acıbadem University, Istanbul, Turkey; i Urology Department, Fundació Puigvert, Universitat Autònoma de Barcelona, Barcelona, Spain; j London, UK; k Skåne University Hospital, Lund University, Lund, Sweden

**Keywords:** Bacillus Calmette-Guérin, Bladder cancer, High-grade non–muscle-invasive bladder cancer, Quality of life

## Abstract

**Background:**

Adverse events induced by intravesical bacillus Calmette-Guérin (BCG) to treat high-grade non–muscle-invasive bladder cancer (NMIBC) often lead to treatment discontinuation. The EAU-RF NIMBUS trial found a reduced number of standard-dose BCG instillations to be inferior with the standard regimen. Nonetheless, it remains important to evaluate whether patients in the reduced BCG treatment arm had better quality of life (QoL) due to a possible reduction in toxicity or burden.

**Objective:**

To evaluate whether patients in the EAU-RF NIMBUS trial experienced better QoL after a reduced BCG instillation frequency.

**Design, setting, and participants:**

A total of 359 patients from 51 European sites were randomized to one of two treatment arms between December 2013 and July 2019. The standard frequency arm (*n* = 182) was 6 weeks of BCG induction followed by 3 weeks of maintenance at months 3, 6, and 12. The reduced frequency arm (*n* = 177) was BCG induction at weeks 1, 2, and 6, followed by maintenance instillations at weeks 1 and 3 of months 3, 6, and 12.

**Outcome measurements and statistical analysis:**

Analyses were performed using an intention-to-treat analysis and a per-protocol analysis. QoL was measured using the European Organization for Research and Treatment of Cancer (EORTC) Quality of Life Questionnaire Core 30 version 3.0 (QLQ-C30 v.03) prior to the first and last instillations of each BCG cycle. Group differences were determined using linear regression corrected for QoL at baseline. Differences in QoL over time were tested for significance using a linear mixed model. Side effects were recorded by the treating physician using a standardized form. Chi-square tests were used to compare the side-effect frequency between the arms.

**Results and limitations:**

There were no significant differences in the means of each QoL scale between the two arms. There were also no significant changes over time in all QoL domains for both arms. However, differences in the incidence of general malaise at T1 (before the last induction instillation), frequency, urgency, and dysuria at T7 (before the last maintenance instillation) were detected in favor of the reduced frequency arm.

**Conclusions:**

Reducing the BCG instillation frequency does not improve the QoL in NMIBC patients despite lower storage symptoms.

**Patient summary:**

In this study, we evaluated whether a reduction in the number of received bacillus Calmette-Guérin instillations led to better quality of life in patients with high-grade non–muscle-invasive bladder cancer. We found no difference in the quality of life between the standard and the reduced bacillus Calmette-Guérin instillation frequency. We conclude that reducing the number of instillations does not lead to better quality of life in patients with high-grade non–muscle-invasive bladder cancer.

## Take Home Message

This study did not find better quality of life with a reduction in the number of bacillus Calmette-Guérin instillations in patients with high-grade non–muscle-invasive bladder cancer. This result together with the previous finding that a reduced frequency schedule is inferior underlines the use of a standard bacillus Calmette-Guérin instillation schedule.

## 1 Introduction

Urothelial bladder cancer (UBC) carries a large global disease burden, being the 11th most common cancer, with approximately 550 000 new cases annually [1]. Nearly 75% of all primary UBC patients are diagnosed with non–muscle-invasive bladder cancer (NMIBC). Patients with high-grade NMIBC have increased risks of recurrence, progression, and metastases [2]. Intravesical bacillus Calmette-Guérin (BCG) instillations following a transurethral resection of the bladder tumor (TURBT) are the standard of care to reduce these risks. The European Association of Urology (EAU) guidelines recommend a weekly instillation for 6 wk as an induction phase, followed by a maintenance phase of 1 yr (three times 3 weekly instillations at 3, 6, and 12 mo) after TURBT for intermediate-risk and up to 3 yr for high-risk patients [3], [4], [5]. Adverse events, however, are significant during the long-term administration of BCG, often leading to treatment discontinuation [6], [7].

The European Organization for Research and Treatment of Cancer (EORTC) trial (EORTC 30962) concluded that BCG dose reduction did not affect toxicity level and led to higher recurrence rates [8]. The European EAU-RF NIMBUS trial evaluated whether a reduced instillation frequency during both the induction and the maintenance phase is noninferior to EAU guideline standard of care [9]. Unfortunately, safety analyses showed the reduced approach to be inferior to the standard approach for the risk of recurrence, leading to early cessation of patient recruitment to avoid further harm in the reduced BCG frequency arm. The current post hoc analysis of the EAU-RF NIMBUS trial evaluated whether patients with reduced BCG instillation frequency in both the induction and the maintenance phase experienced lower toxicity and consequently better quality of life (QoL) than patients receiving the standard BCG instillation frequency.

## 2 Patients and methods

The EAU-RF NIMBUS trial was a European randomized controlled trial that assessed whether a reduction in the BCG instillation frequency is noninferior to the standard BCG frequency in patients with high-grade NMIBC (Ta-T1) [9]. Recruitment took place between December 2013 and July 2019 at 51 study sites spread across Germany, The Netherlands, France, Belgium, and Spain. Patient recruitment was ceased on July 1, 2019, after a data review and safety analysis by the Independent Data Monitoring Committee (IDMC) showed the reduced BCG instillation arm to be inferior to the standard BCG instillation arm with regard to the risk of recurrence.

The trial had been approved by all the relevant institutional review boards and independent ethics committees, and had been performed according to the Declaration of Helsinki [10], Good Clinical Practice, and local regulatory requirements.

### 2.1 Inclusion and exclusion criteria

BCG-naïve patients who had been clinically diagnosed with primary or recurrent high-grade NMIBC (Ta or T1), with single or multiple urothelial papillary bladder carcinoma(s), and with or without concomitant carcinoma in situ (CIS) were eligible. A routine repeated TURBT (re-TUR and/or re-re-TUR) had to be performed to confirm the absence of muscle-invasive cancer. High-grade Ta patients were allowed to be included without a re-TUR in case a biopsy specimen confirmed the complete removal of the tumor and included detrusor muscle tissue.

The exclusion criteria were having had previous systemic or multi-instillation intravesical chemotherapy within the preceding 3 mo, having any type of tumor(s) in the upper urinary tract or prostatic urethra at any time, having any immunodeficiency, and having any other type of malignancy besides basal cell carcinoma of the skin or localized prostate cancer under active surveillance.

### 2.2 Randomization

After enrolment, patients were allocated using a validated randomization program (EAU-RF website) according to the minimization method with a random element as described by Pocock [11]. Stratification factors included center, Ta versus T1, concomitant CIS versus no CIS, single versus multiple tumors, and BCG strain (Connaught, Medac, or Tice). The patients were randomized to either one of two treatment groups:

1. The standard frequency (SF) arm. Induction: once a week BCG instillations at weeks 1–6; maintenance: once a week instillations at weeks 1–3 at months 3, 6, and 12 (15 planned instillations).
2. The reduced frequency (RF) arm. Induction: once a week BCG instillations at weeks 1, 2, and 6; maintenance: instillations at weeks 1 and 3 at months 3, 6, and 12 (9 planned instillations).

Follow-up was conducted through cystoscopy and urine cytology every 3 mo during the first 2 yr and every 6 mo thereafter. Histological confirmation had to be provided in case of CIS, or if there was a suspicion of disease recurrence.

Patients’ participation in the study was ended in case of a recurrence in the bladder, a urothelial carcinoma in the upper urinary tract or prostatic urethra, or presence of distant metastases, or in case systemic chemotherapy was indicated.

### 2.3 Questionnaires

**Fig. 1:**
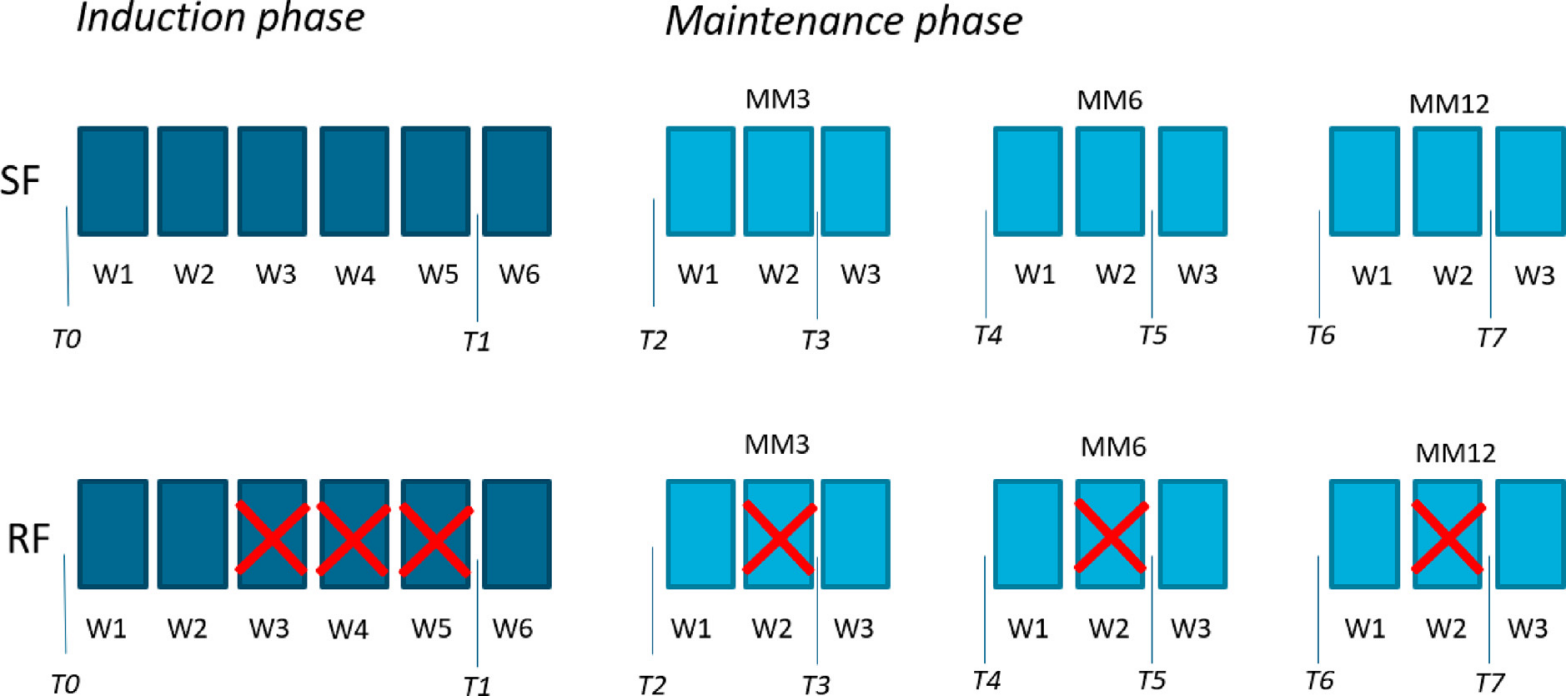
Overview of the two treatment arms where each block represents a BCG instillation. The crossed out blocks in the RF arm represent the instillations that had not been performed. The different time points represent the moments the QLQ-C30 questionnaire and the side-effect evaluations had been completed. BCG = bacillus Calmette-Guérin; MM = maintenance month; QLQ-C30 = Quality of Life Questionnaire Core 30 version 3.0; RF = reduced frequency; SF = standard frequency; T = time point; W = week.

QoL in patients was determined using the EORTC Quality of Life Questionnaire Core 30 version 3.0 (QLQ-C30 v3.0) [12]. This validated questionnaire consists of 30 items that are primarily scored on a 4-point Likert scale: 1, “not at all”; 2, “a little”; 3, “quite a bit”; and 4, “very much.” It includes five functional scales (physical, role, cognitive, emotional, and social), three symptom scales (pain, fatigue, and nausea/vomiting), experienced financial disease impact, and a global health status. The remaining items evaluate any additional symptoms that are commonly perceived in cancer patients (dyspnea, appetite loss, sleep disturbance, constipation, and diarrhea). Paper questionnaires on QoL were handed out during an outpatient visit at the right time points. The questionnaires were completed prior to the first and the last instillation of each BCG cycle, leading to a total of eight measurement points (T0–T7; see Fig. 1). The endpoint of the NIMBUS trial was time to first recurrence. Consequently, QoL questionnaires were not filled out anymore if patients experienced a recurrence.

In addition, treating physicians were responsible for carrying out side effect (SE) evaluations by means of a form that included known local and systemic SEs (World Health Organization grading of toxicity: grade 1, mild; grade 2, moderate; grade 3, severe; and grade 4, life-threatening toxicity) prior to the first and the last instillation of each BCG cycle [13].

### 2.4 Endpoints

The primary endpoint for the analysis was QoL. Additionally, toxicity incidence and severity were recorded.

### 2.5 Statistical analyses

All statistical analyses were performed at a 5% significance level (*p* < 0.05) using the IBM SPSS version 26 (IBM Corporation, Armonk, NY, USA). Intention-to-treat (ITT) QoL and SE analyses were performed for time points T1 (induction week 6), T5 (maintenance month 6, week 3), and T7 (maintenance month 12, week 3).

All the five functional and three symptom scales plus the individual symptom items of the questionnaires were transformed to a 0–100 score. A high scale score represents a higher response level. Thus, a high score for a functional scale represents a high/healthy level of functioning, a high score for the global health status/QoL represents high QoL, but a high score for a symptom scale/item represents a high level of symptoms/problems. Differences in the mean QoL between the two treatment arms were evaluated using linear regression at T1, T5, and T7 while adjusting for T0 (baseline measurement, ie, prior to induction week 1). Differences between the trends in QoL of the two treatment arms were tested for significance by performing a linear mixed model using time as the fixed factor with eight levels (T0–T7). Chi-square or Fisher exact tests were used to test for significant differences in the number of SEs between the two treatment arms.

After performing the ITT QoL and SE analyses, supplementary per-protocol (PP) QoL and SE analyses were performed. Patients were excluded from the PP analysis if they had incomplete treatment due to missed instillations, had extra BCG instillation(s), switched treatment arm after the study’s premature stop, or stopped treatment for other reasons besides SEs or recurrence.

## 3 Results

**Fig. 2:**
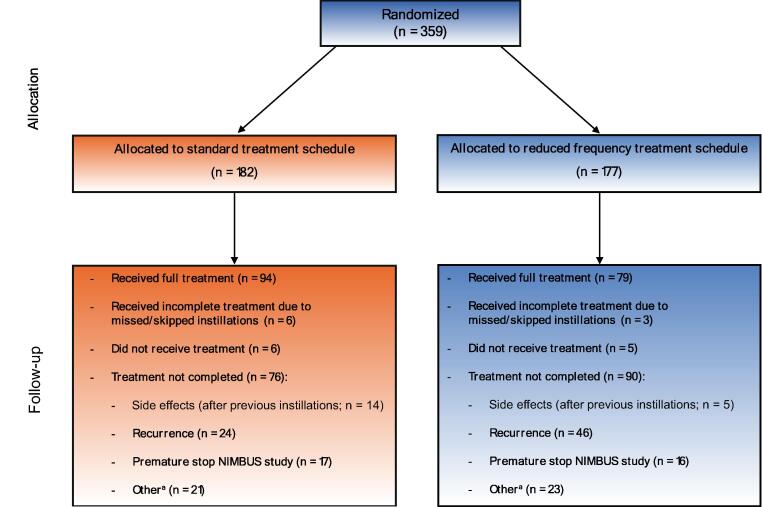
Intravesical treatments received and reasons to stop. a Examples of “Other” are consent withdrawn, lost to follow-up, and patient not compliant.

A total of 359 patients were randomized to one of the two treatment arms. The SF arm contained 182 patients, while the RF arm contained 177 patients. At baseline, there were no significant differences in characteristics between the two treatment arms (Table 1). At the time of study discontinuation, 52% (*n* = 94) of the patients in the SF arm received all 15 planned instillations. In total, 48 (26%) patients in this arm received nine or fewer instillations. In the RF arm, 45% (*n* = 79) received all nine planned instillations at the time of study stop. In the SF arm, 24 patients developed a recurrence or new CIS within 1 yr and went off study. In the RF arm, this number was 46 (Fig. 2). In total, 30 and 55 patients in the SF and RF arms, respectively, developed a recurrence.

**Table 1:** Baseline characteristics stratified by the two treatment arms

Characteristic	Overall cohort (*n* = 359)	Standard frequency arm (*n* = 182)	Reduced frequency arm (*n* = 177)	*p* value
Age, mean (95% CI)	70.8 (69.9–71.8)	70.9 (69.5–72.2)	70.8 (69.4–72.1)	0.93
Sex, n (%)				0.59
Male	296 (82.5)	152 (83.5)	144 (81.4)	
Female	63 (17.6)	30 (16.5)	33 (18.6)	
Ethnicity, n (%)				0.75
Black	1 (0.3)	1 (0.6)	0 (0.0)	
Caucasian	300 (83.6)	150 (82.4)	150 (84.8)	
Oriental	2 (0.6)	1 (0.6)	1 (0.6)	
Other	35 (9.7)	19 (10.4)	16 (9.0)	
Missing	21 (5.8)	11 (6.0)	10 (5.6)	
Stage, n (%)				0.21
Tis	3 (1.7)^a^	3 (1.7)^a^	0 (0)	
Ta	162 (45.1)	80 (44.0)	82 (46.3)	
T1	197 (54.9)	102 (56.0)	95 (53.7)	
T2	1 (0.6)^b^	0 (0)	1 (0.6)^b^	
Grade, n (%)				0.50
Low grade	2 (0.6)	2 (1.1)	0 (0.0)	
High grade	357 (99.4)	180 (98.9)	177 (100)	
Presentation, n (%)				0.91
Primary	330 (91.9)	167 (91.7)	163 (92.1)	
Recurrent	29 (8.1)	15 (8.2)	14 (7.9)	
No. of tumors, n (%)				0.38
Single	201 (56.0)	106 (58.2)	95 (53.7)	
Multiple	158 (44.0)	76 (41.8)	82 (46.3)	
Previous intravesical therapy, n (%)				0.67
None	348 (96.9)	177 (97.3)	171 (96.6)	
BCG^c^	1 (0.3)	1 (0.6)	0 (0.0)	
Doxorubicin	2 (0.6)	1 (0.6)	1 (0.6)	
Mitomycin	8 (2.2)	3 (1.7)	5 (2.8)	
BCG strain, n (%)				0.49
Connaught	7 (1.9)	2 (1.1)	5 (2.8)	
Medac	320 (89.1)	164 (90.1)	156 (88.1)	
Tice	32 (8.9)	16 (8.8)	16 (9.0)	

BCG = bacillus Calmette-Guérin; CI = confidence interval; CIS = carcinoma in situ.

a Three patients had CIS only: 1× treatment completed (15 instillations), patient included in follow-up, no recurrence; 1× treatment completed (14 instillations), patient included in follow-up, first recurrence, and tumor in prostatic urethra at month 36; 1× consent withdrawn after six instillations, patient included in follow-up until that time point, no recurrence.

b Patient did not receive BCG and was not included in follow-up.

c One patient was previously treated with BCG. This was a protocol violation. The patient was kept in the analyses for consistency with the original paper on the NIMBUS trial (by Grimm et al. [9]).

### 3.1 QoL analyses

**Fig. 3:**
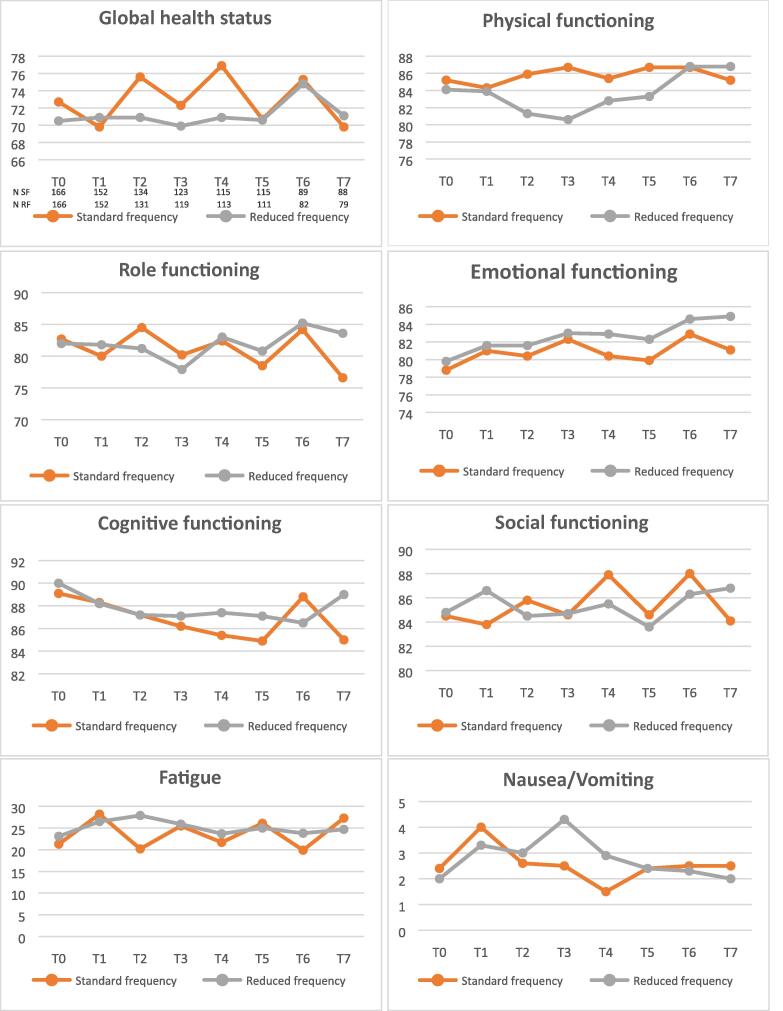

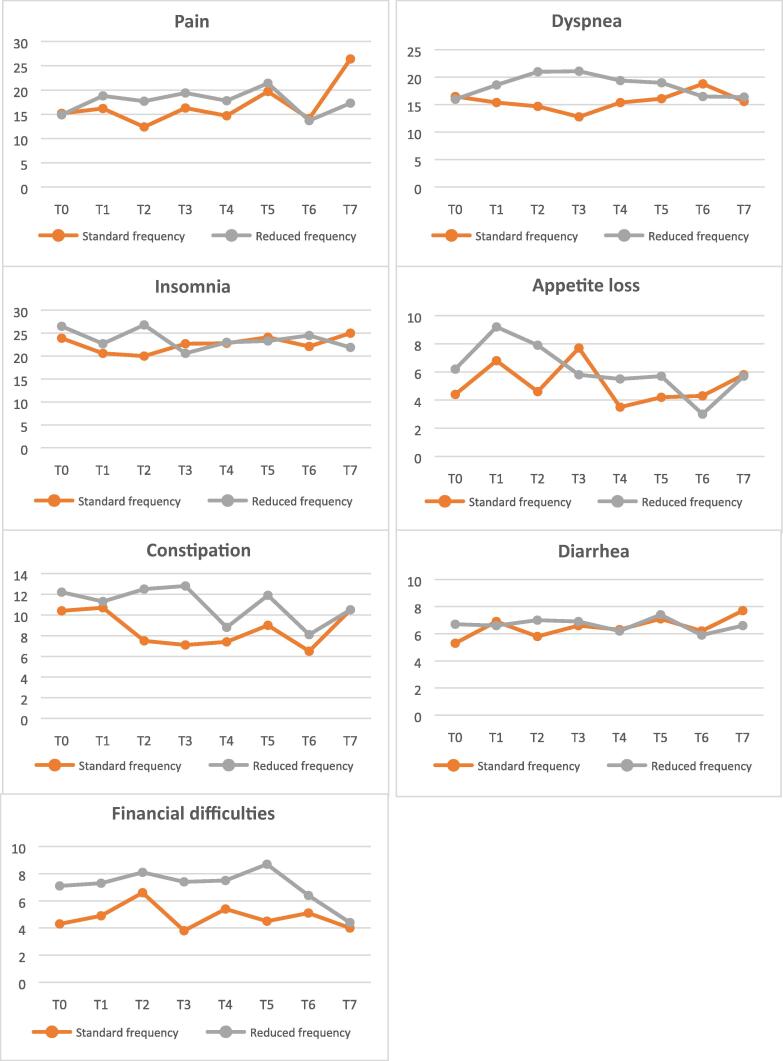
Summary of the results from the EORTC QLQ-C30 where the *X* axes represent the time points and the *Y* axes represent the mean QoL of the different EORTC scales and items (all scales have a range 0–100; for QoL scales, a higher score means better QoL; for symptom scales, a higher score means more symptoms). EORTC = European Organization for Research and Treatment of Cancer; QoL = quality of life; QLQ-C30 = Quality of Life Questionnaire Core 30 version 3.0; RF = reduced frequency; SF = standard frequency.

The QLQ-C30 questionnaires were completed by 304 (84.7%) patients at T1, 226 (63.0%) patients at T5, and 168 (47.2%) patients at T7. Detailed results of the questionnaires can be found in Table 2. A summary of the results is depicted in Figure 3. Aside from the physical functioning at T5 (*p* = 0.05), we found no differences in the means of any QoL scale between the two treatment arms (*p* > 0.05; Table 2). Moreover, the linear mixed model, which was adjusted for T0, did not show any statistically significant temporal changes in any QoL domain for both the SF and the RF arm (91% in the SF arm and 94% in the RF arm completed the QoL assessment at T0).

**Table 2:** Results of the EORTC QLQ-C30 scale at T1 (induction week 6), T5 (maintenance month 6, week 3), and T7 (maintenance month 12, week 3)

EORTC scale	T1 (*n* = 304)			T5 (*n* = 226)			T7 (*n* = 168)		
	SF (*n* = 152) Mean (SD)	RF (*n* = 152) Mean (SD)	*p* value	SF (*n* = 119) Mean (SD)	RF (*n* = 107) Mean (SD)	*p* value	SF (*n* = 92) Mean (SD)	RF (*n* = 76) Mean (SD)	*p* value
Global health status/QoL	69.8 (19.3)	70.9 (21.4)	0.85	70.7 (21.7)	70.6 (23.7)	0.96	69.8 (23.2)	71.1 (24.2)	0.59
Functional scales									
Physical functioning	84.3 (19.3)	83.9 (18.7)	0.57	86.7 (17.5)	83.3 (17.8)	**0.05**	85.2 (18.2)	86.8 (16.0)	0.49
Role functioning	80.0 (27.2)	81.8 (26.7)	0.87	78.5 (28.0)	80.8 (26.0)	0.37	76.6 (28.8)	83.6 (23.2)	0.45
Emotional functioning	81.0 (21.0)	81.6 (21.4)	0.90	79.9 (23.8)	82.3 (23.8)	0.53	81.1 (20.4)	84.9 (21.0)	0.80
Cognitive functioning	88.3 (16.8)	88.2 (18.8)	0.55	84.9 (20.4)	87.1 (19.5)	0.80	85.0 (22.9)	89.0 (18.2)	0.72
Social functioning	83.8 (23.2)	86.6 (21.0)	0.77	84.6 (20.8)	83.6 (24.9)	0.24	84.1 (21.1)	86.8 (22.7)	0.22
Symptom scales/items									
Fatigue	28.2 (25.7)	26.5 (25.8)	0.75	26.1 (24.4)	25.0 (25.0)	0.34	27.3 (24.1)	24.7 (25.0)	0.97
Nausea/vomiting	4.0 (12.4)	3.3 (9.2)	0.38	2.4 (6.6)	2.4 (7.4)	0.36	2.5 (10.8)	2.0 (5.4)	0.30
Pain	16.2 (23.3)	18.8 (26.5)	0.99	19.7 (27.0)	21.4 (26.9)	0.09	26.4 (30.6)	17.3 (24.0)	0.75
Dyspnea	15.4 (25.4)	18.6 (27.3)	0.25	16.1 (25.7)	19.0 (28.3)	0.19	15.6 (25.9)	16.4 (23.5)	0.90
Insomnia	20.6 (29.5)	22.7 (27.6)	0.78	24.1 (28.8)	23.3 (30.2)	0.53	25.0 (28.7)	21.9 (30.6)	0.76
Appetite loss	6.8 (16.9)	9.2 (21.1)	0.49	4.2 (11.1)	5.7 (16.2)	0.62	5.8 (16.1)	5.7 (14.8)	0.87
Constipation	10.7 (22.3)	11.3 (23.1)	1.00	9.0 (21.6)	11.9 (21.7)	0.71	10.5 (22.6)	10.5 (21.2)	0.69
Diarrhea	6.9 (16.1)	6.6 (15.4)	0.23	9.0 (20.7)	5.3 (14.6)	0.87	7.7 (15.8)	6.6 (14.4)	0.96
Financial difficulties	4.9 (15.1)	7.3 (20.0)	0.34	4.5 (14.3)	8.7 (22.6)	0.78	4.0 (12.0)	4.4 (13.7)	0.57

EORTC QLQ-C30 = European Organization for Research and Treatment of Cancer Quality of Life Questionnaire Core 30 version 3.0; QoL = quality of life; RF = reduced frequency; SD = standard deviation; SF = standard frequency.

### 3.2 Toxicity

SE evaluations were completed in 57.7% of patients at T1, 44.5% of patients at T5, and 34.6% of patients at T7 (Table 3). For patients for whom an SE form was not filled out, we conducted an enquiry among the participating urologists. Thirty-three of 51 sites responded; 26 out of the 33 responding urologists (79%) stated that there were no SEs when the SE form was not filled out. In patients recruited by the remaining seven sites (21%), there might have been SEs, but the grading was not assessed.

**Table 3:** Incidence of WHO grade I–IV side effects^a^ by treatment groups at time points T1 (induction week 6), T5 (maintenance month 6, week 3), and T7 (maintenance month 12, week 3)

Side effect	T1			T5			T7		
	Standard frequency (*n* = 182) *N* (%)	Reduced frequency (*n* = 177) *N* (%)	*p* value	Standard frequency (*n* = 182) *N* (%)	Reduced frequency (*n* = 177) *N* (%)	*p* value	Standard frequency (*n* = 182) *N* (%)	Reduced frequency (*n* = 177) *N* (%)	*p* value
No SE form filled out^b^	78 (42.9)	74 (41.8)	–	96 (52.7)	104 (58.8)	–	116 (63.7)	120 (67.8)	–
Local SEs									
No local SEs reported (grade 0)	44 (24.1)	56 (31.6)	–	31 (17.1)	41 (22.6)	–	18 (9.9)	34 (19.3)	–
Total no. of patients with SEs	60 (33.0)	47 (26.6)	0.08	55 (30.2)	33 (18.6)	**0.01**	48 (26.4)	23 (12.9)	**<0.001**
Frequency	37 (20.3)	29 (16.4)	0.25	39 (21.4)	25 (14.1)	0.14	33 (18.1)	13 (7.3)	**0.002**
Urgency	44 (24.2)	30 (16.9)	**0.05**	38 (20.9)	24 (13.6)	0.13	30 (16.5)	14 (7.9)	**0.02**
Dysuria	24 (13.2)	15 (8.5)	0.12	20 (11.0)	16 (9.0)	0.80	25 (13.7)	7 (4.0)	**0.001**
Incontinence	14 (7.7)	14 (7.9)	0.99	8 (4.4)	9 (5.1)	0.56	5 (2.7)	7 (4.0)	0.38
Macroscopic hematuria	15 (8.2)	10 (5.6)	0.30	17 (9.3)	14 (7.9)	0.89	12 (6.6)	7 (4.0)	0.37
Bacterial cystitis	3 (1.6)	8 (4.5)	0.12	5 (2.7)	2 (1.1)	0.34	3 (1.6)	2 (1.1)	0.77
Chemical cystitis	5 (2.7)	3 (1.7)	0.48	4 (2.2)	5 (2.8)	0.56	5 (2.7)	0 (0)	**0.03**
Other	4 (2.2)	6 (3.4)	0.51	5 (2.7)	1 (0.6)	0.14	2 (1.1)	0 (0)	0.19
Total no. of SEs	146	115	0.13^c^	136	96	0.09^c^	115	50	**<0.001**^c^
Systemic SEs									
No systemic SEs reported (grade 0)	78 (43.4)	86 (48.6)	–	61 (33.6)	61 (33.9)	–	53 (29.2)	51 (28.8)	–
Total no. of patients with SEs	25 (13.7)	17 (9.6)	0.17	25 (13.7)	13 (7.3)	0.09	13 (7.1)	6 (3.4)	0.16
Fever	5 (2.7)	11 (6.2)	0.11	10 (5.5)	8 (4.5)	0.87	3 (1.6)	4 (2.3)	0.56
General malaise	16 (8.8)	6 (3.4)	**0.03**	17 (9.3)	7 (3.9)	0.07	9 (4.9)	4 (2.3)	0.23
Skin rash	1 (0.5)	2 (1.1)	0.56	0	0	–	0	0	–
BCG-induced lung infection	0	0	–	0	0	–	0	0	–
Sepsis	1 (0.5)	1 (0.6)	1.00	2 (1.1)	0	0.19	0	0	–
Other	5 (2.7)	2 (1.1)	0.25	7 (3.8)	1 (0.6)	0.05	0 (0)	2 (1.1)	0.19
Total no. of SEs	28	22	0.21^c^	36	16	0.07^c^	14	8	0.19^c^

BCG = bacillus Calmette-Guérin; RF = reduced frequency; SE = side effect; SF = standard frequency; WHO = World Health Organization.

a The following grade 3 or grade 4 side effects were observed: (1) grade 3 local side effects: one event in the RF group (M3W1) and seven events in four patients in the SF group (3× M2W6, 2× M6W1, and 2× M6W3); (2) grade 4 local side effects: none; (3) grade 3 systemic side effects: one event in the RF group (M3W1); and (4) grade 4 systemic side effects: seven events in four patients in the SF group (M6W1, M6W3, and M12W2).

b For patients for whom a side-effect form was not filled out, we assumed that there were no side effects.

c Calculated using Mann-Whitney *U* test based on the average number of side effects per patient.

In Table 3, we present a best case scenario assuming that there were no SEs in patients in whom the SE form was not filled out. Globally, the treatment toxicity did not exceed grade II in the majority of the patients. Grade III and IV local SEs were more frequent in the SF arm (*n* = 7; 3.8%) than in the RF arm (*n* = 1; 0.6%; *p* = 0.07; data not shown). The number of grade III and IV systemic SEs were similar to that of the local SEs (3.8% and 0.6%, respectively).

Overall, local SEs were reported more often than systemic SEs at all time points for both arms. Urination problems (frequency, urgency, dysuria, and incontinence) were the most commonly reported local SEs, whereas fever and general malaise were the most frequent systemic SEs. Although mostly insignificant, the numbers of recorded local and systemic SEs were generally higher in the SF arm. We found the SF arm to have a significantly higher frequency of total local SEs at T7 (*p* ≤ 0.001). Moreover, the total number of patients with local SEs was significantly higher in the SF arm than in the RF arm at T5 (*p* = 0.01) and T7 (*p* ≤ 0.001). Specifically, there were significant differences in the incidence of urgency (*p* = 0.05) and general malaise (*p* = 0.03) at T1, and frequency (*p* = 0.002), urgency (*p* = 0.02), dysuria (*p* = 0.001), and chemical cystitis (*p* = 0.03) at T7 favoring RF arm patients.

When a worst case scenario is assumed in which all patients without an SE grading form had SEs, we see a prevalence of local SEs at T2 of 42% and 35% in the SF and RF arms, respectively. At T5 and T7, the prevalence was 41.2% versus 31.1% and 39.6% versus 27.1%, respectively. The prevalence of systemic SEs at T2, T5, and T7 was 22.5% versus 18.1%, 24.7% versus 19.8%, and 20.3% versus 17.5%, respectively. Overall, we see the same pattern of fewer SEs in the RF arm than in the SF arm, but differences are small.

### 3.3 PP analyses

After excluding patients according to the PP criteria, a total of 249 patients remained, of whom 123 (49.4%) were randomized to the SF arm and 126 (50.6%) to the RF arm. No significant differences in the baseline characteristics were observed between the two arms (Supplementary Table 1). Supplementary Table 2 and Supplementary Figure 1 summarize the results obtained from the QoL analyses. For the largest part, similar results to those in the ITT analysis are seen. The difference in physical functioning in the ITT analysis at T5 is no longer present in the PP results. However, we found a higher mean score of diarrhea in the RF arm at T1 (*p* = 0.01), which was not the case in the ITT analysis. Again, the linear mixed model did not display statistically significant temporal changes in any QoL domain for both the SF and the RF arm (*p* > 0.05). The PP analysis of SEs was also largely consistent with the ITT analysis (Supplementary Table 3). However, unlike in the ITT analysis, no significant differences were found in the total number of patients with local SEs at T5 (*p* = 0.15), urgency at T1 (*p* = 0.39), and general malaise at T1 (*p* = 0.06). We additionally found the number of patients with bacterial cystitis in the SF arm to be significantly higher than that in the RF arm (*p* = 0.03), which was not the case in the ITT analysis.

## 4 Discussion

An analysis of EAU-RF NIMBUS study data did not show better QoL in patients undergoing an RF BCG instillation regimen. However, there were significant differences in the incidence of general malaise at T1, and of storage symptoms of frequency, urgency, and dysuria at T7 favoring RF arm patients. Previous studies showed contrasting results in terms of the QoL and toxicity experienced after a dosage reduction in BCG. Yokomizo et al. [14] found a lower BCG dose (40 mg) to be associated with lower toxicity and better QoL than the standard BCG dose (80 mg). This study focused primarily on an eight-instillation induction phase, while QoL was assessed only once after the induction phase had ended. The EORTC 30962 trial analyzed the efficacy of one-third BCG doses compared with the standard dose. They did not report any difference in toxicity between the reduced and full-dose arms [8]. This trial, however, was mainly designed to analyze the toxicity after the maintenance phase and did not focus on the induction phase. Nonetheless, these studies focused on the effect of a reduced dose of BCG instillations, whereas our study focused on a full dose but RF of BCG instillations. A direct comparison of these studies is therefore difficult.

In accordance with literature, our study reported urinary SEs, general malaise, and fever as the most frequent SEs caused by BCG instillations [15], [16]. These SEs were predominantly mild to moderate, reflecting generally good BCG tolerability.

A reduced BCG instillation frequency however significantly decreased the number of overall SEs. This difference was significant for general malaise (*p* = 0.03) at T1, and for frequency (*p* = 0.002), urgency (*p* = 0.02), and dysuria (*p* = 0.001) at T7. In fact, three times more patients in the SF arm did not complete the instillations due to SEs (14 vs 5 patients).

Interestingly, the higher toxicity reported in the SF arm did not translate into worse QoL. This unexpected result may be explained by the instrument used to measure QoL (QLQ-C30), which is not optimal for this group of patients. Although the QLQ-C30 questionnaire has been validated internationally, it does not focus directly on (non–muscle-invasive) bladder cancer (BC). Literature suggests that the questionnaire fails to assess finer BC-specific details/domains, which reduces the responsiveness to changes. Domains such as sexual functioning, self-consciousness, embarrassment, and psychological distress are of greater importance in BC patients but are not assessed thoroughly by the QLQ-C30 questionnaire [17]. Several BC-specific questionnaires have been designed to offer an instrument that closes these gaps, such as the EORTC QLQ-NIMBC24 questionnaire, which has shown excellent measurement properties with regard to validity, reliability, and responsiveness [18], but this did not exist at the time of the original trial design.

The randomized setting of our study is a strength. In addition, the eight time point QoL evaluations over a year should have been able to pick up temporal QoL changes. Nonetheless, there are limitations to this post hoc analysis. In addition to the suboptimal QoL questionnaire, QoL was not measured anymore after the endpoint (a recurrence) was reached so that the influence of, for example, extra TURBTs could not be studied. Moreover, the large number of unanswered questionnaires resulted in a smaller number of evaluable patients (again raising further questions about the suitability of the instrument used to measure QoL changes). Lastly, the patients could not be blinded to RF instillation, which may have induced a response bias. This may thus have instigated some sort of placebo effect in patients to indeed experience better QoL with RF BCG instillations and vice versa.

## 5 Conclusions

An analysis of the EAU-RF NIMBUS study data did not show better QoL with EORTC QLQ-C30 v3.0, in patients undergoing an RF BCG instillation regimen despite lower storage symptoms at T7 in favor of RF. This finding may possibly be explained by the insensitivity of the EORTC QLQ-C30 questionnaire for small QoL domain changes. Our study, together with the previous finding that an RF schedule is inferior, supports the use of a standard BCG instillation schedule.

  ***Author contributions:*** Lambertus A.L.M. Kiemeney had full access to all the data in the study and takes responsibility for the integrity of the data and the accuracy of the data analysis.

  *Study concept and design*: Caris, Grimm, Colombel, Babjuk, Türkeri, Palou, Patel, Witjes, van der Heijden, Kiemeney.

*Acquisition of data*: Grimm, Colombel, Muilwijk, Martínez-Piñeiro, Babjuk, Türkeri, Palou, Patel, Bjartell, Caris, Witjes, van der Heijden.

*Analysis and interpretation of data*: van Straten, Caris, van der Heijden, Kiemeney

*Drafting of the manuscript*: van Straten, van der Heijden, Kiemeney

*Critical revision of the manuscript for important intellectual content*: van Straten, Caris, Grimm, Colombel, Muilwijk, Martínez-Piñeiro, Babjuk, Türkeri, Palou, Patel, Bjartell, Witjes, van der Heijden, Kiemeney.

*Statistical analysis*: van Straten, Caris, Kiemeney.

*Obtaining funding*: Grimm, Colombel, Witjes.

*Administrative, technical, or material support*: Caris, Witjes.

*Supervision*: Grimm, Witjes, van der Heijden, Kiemeney.

*Other*: None.

  ***Financial disclosures:*** Lambertus A.L.M. Kiemeney certifies that all conflicts of interest, including specific financial interests and relationships and affiliations relevant to the subject matter or materials discussed in the manuscript (eg, employment/affiliation, grants or funding, consultancies, honoraria, stock ownership or options, expert testimony, royalties, or patents filed, received, or pending), are the following: Luis Martínez Piñeiro discloses grants from Comunidad de Madrid—IMMUNOTHER CAN-CM (B2017/BMD3733), during the conduct of the study. Marko Babjuk reports grants from Czech Health Grand Agency (AZV), studies supported by Hamlet Pharma and Sotio, an advisory role for Ferring, and speaker tasks for Astellas, Ferring, and Ipsen. Anup Patel discloses consulting or advisory role, travel, accommodations, and expenses from Pfizer. Anders S. Bjartell discloses consulting or advisory role for Astellas, Bayer, and Janssen; being in the speakers’ bureau from Astellas, Bayer, Ferring, and Janssen; research funding from Ferring, Astellas, and Bayer; and travel, accommodations, and expenses from Astellas, Bayer, Janssen, and Ferring. Marc Oliver Grimm reports grants and personal fees from Novartis and BMS; and personal fees from Pfizer, Bayer HealthCare, Astellas, Intuitive Surgical, Sanofi Aventis, Hexal, Apogepha, Amgen, AstraZeneca, MSD, Janssen Cilag, Ono Pharma, Ipsen Pharma, Medac, and Merck, outside the submitted work. Antoine van der Heijden, Marc Colombel, Tim Muilwijk, Levent N. Türkeri, Joan Palou, Christien Caris, Christine van Straten, Wim P.J. Witjes, and Lambertus Kiemeney have nothing to disclose.

  ***Funding/Support and role of the sponsor:*** This work was supported by Deutsche Krebshilfe (DKH 109724); MEDAC (provision of BCG Medac in the RF arm in Germany); Department of Urology, Hospital Edouard Herriot, Lyon, France; Department of Urology, Jena University Hospital, Jena, Germany; and EAU Research Foundation.

  ***Acknowledgments:*** We thank all the NIMBUS trial participants and researchers of the EAU Research Foundation NIMBUS Study Group for their contributions. The members of the Study group are: Germany: Dr. Jörg Horstmann, Aachen; Dr. Stefan Machtens, Bergisch Gladbach; Dr. Eberhard Mumperow, Langenfeld; Dr. Andreas Al Ghazal, Ulm; Dr. Thomas Pulte, Würselen; Dr. Michael Stephan-Odenthal, Leverkusen; Dr. Georgios Gakis, Tübingen; Dr. Mario Kramer, Lübeck; Professor Dr. Marc Oliver Grimm, Jena; Professor Dirk Zaak, Traunstein; Professor Dr. Bernd Schmitz-Dräger, Nürnberg; Dr. Holger Schreier Braunschweig; Dr. Jan Lehmann, Kiel; Dr. Torsten Werner, Herzberg am Harz; Dr. Jörg Klier, Köln; Dr. Jan Marin, Kempen; Dr. Wolfgang Rulf, Erkrath; Dr. Eva Hellmis, Duisburg; Dr. Andreas Schneider, Salzhausen; Dr. Spiegel Halder, Mettmann; Professor Dr. Manfred Wirth, Dresden; Professor Dr. Theodor Klotz, Weiden; Dr. Henrik Suttmann, Hamburg; Dr. Michael Siebels, München-Pasing; Dr. Gerd Rodemer, Wilhelmshaven; Dr. Robert Rudolph, Kirchheim/Teck; and Dipl. med. Roger Zillmann, Berlin-Pankow. The Netherlands: Dr. M. de Bruin, Roermond; Dr. S. Bos, Alkmaar; Professor Dr. R. van Moorselaar, Amsterdam; Professor Dr. T de Reijke, Amsterdam; Dr. J. Boormans, Rotterdam; Dr. B. Wijsman, Tilburg; Dr. H.H.E. van Melick, Nieuwegein; Dr. E van Boven, Beugen; Dr. R.P. Meijer, Utrecht; Dr. A.G. van der Heijden, Nijmegen; Dr. H. Vergunst, Nijmegen; Dr. E. te Slaa, Zwolle/Meppel; and Dr. A.M. Leliveld Kors, Groningen/Winschoten. France: Professor Marc Colombel, Lyon; Professor Alain Ruffion, Pierre-Bénite; Dr. Christian Pfister, Rouen; Professor Morgan Roupret, Paris; Professor Jacques Irani, Le Kremlin-Bicêtre; and Dr. Gabriel Stoica, Alençon. Belgium: Dr. Siska Van Bruwaene, Kortrijk; Dr. Filip Ameye, Gent; Dr. Harm Arentsen, Brugge; and Professor Dr. Steven Joniau, Leuven. Spain: Pastora Beardo, Vitoria Gasteiz (Álava). In addition, we acknowledge the Data Monitoring Committee: Professor Dr. Markus Kuczyk, Professor Dr. George Thalmann, and Professor Dr. Lambertus Kiemeney.
