# Supplementary material for: Quality of Life in Patients with High-grade Non–muscle-invasive Bladder Cancer Undergoing Standard Versus Reduced Frequency of Bacillus Calmette-Guérin Instillations: The EAU-RF NIMBUS Trial
Source: Eur Urol Open Sci. 2023 Sep 12;56:15–24. doi: 10.1016/j.euros.2023.08.004 (PMC10562176; doi:10.1016/j.euros.2023.08.004)
Supplement: Supplementary data 1 [file mmc1.pdf]

Supplementary information to **Quality of Life in Patients with High-grade Non–muscle-invasive Bladder Cancer Undergoing Standard Versus Reduced Frequency of Bacillus Calmette-Guérin Instillations: The EAU-RF NIMBUS Trial**

Supplementary Figure 1

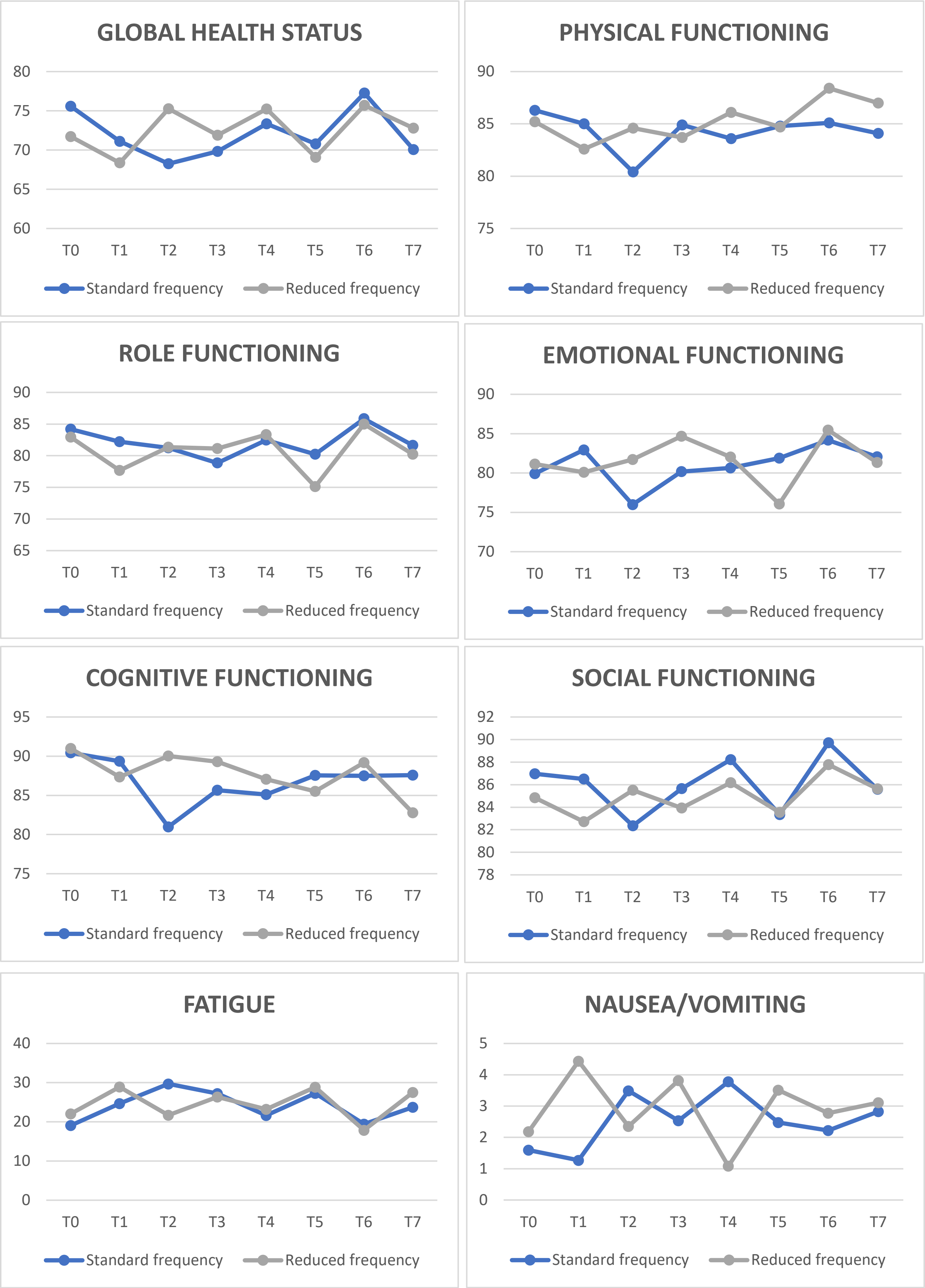

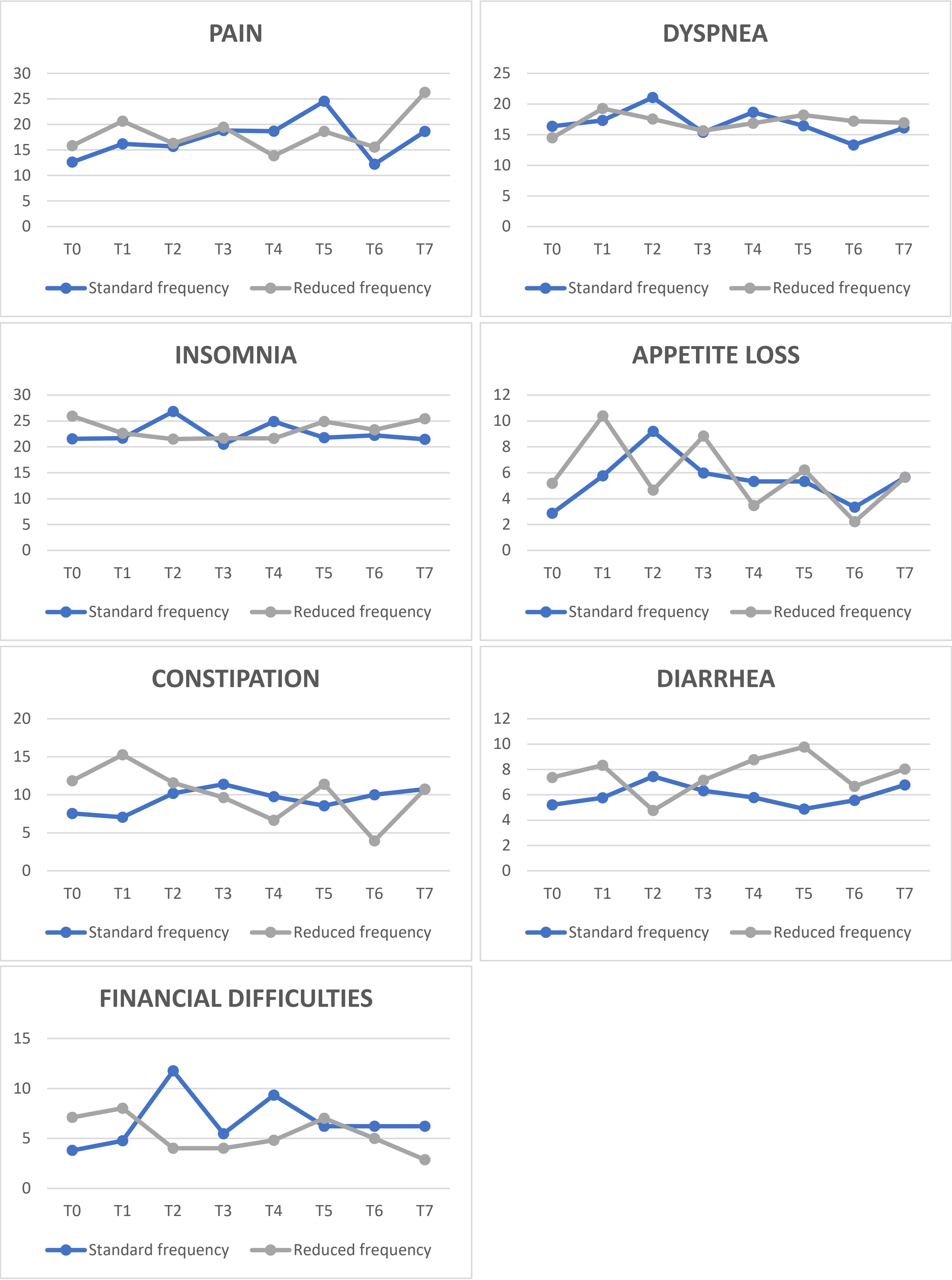

Supplementary Figure 1. Per Protocol analysis: Summary of the results from the EORTC QLQ-C30 where the X-axes represent the time points and the Y-axes represent the mean QoL of the different EORTC scales and items (all scales have a range 0-100; for QoL scales a higher score means better QoL; for symptom scales, a higher score means more symptoms.

Supplementary Table 1. Baseline characteristics stratified by the two treatment arms in a Per Protocol analysis.

Characteristic	**Overall cohort (n=249)**	**Standard frequency arm (n=123)**	**Reduced frequency arm (n=126)**	**p-value**
Age, mean (95%CI)				0.83
	70.6 (70.0-71.2)	70.5 (69.7-71.30)	70.7 (69.9-71.5)	
Sex, n(%)				0.60
Male	197 (79.1)	99 (80.5)	98 (77.8)	
Female	52 (20.9)	24 (19.5)	28 (22.2)	
Ethnicity, n(%)				0.70
Black	-	-	-	
Caucasian	213 (85.5)	102 (82.9)	111 (88.1)	
Oriental	2 (0.8)	1 (0.8)	1 (0.8)	
Other	19 (7.6)	11 (8.9)	8 (6.3)	
Missing	15 (6.0)	9 (7.3)	6 (4.8)	
Stage, n(%)				0.95
Tis	-	-	-	
Ta	113 (45.4)	52 (42.3)	61 (48.4)	
T1	136 (54.6)	71 (57.7)	65 (51.6)	
T2	-	-	-	
Presentation, n(%)				0.51
Primary	230 (92.4)	115 (93.5)	115 (91.3)	
Recurrent	19 (7.6)	8 (6.5)	11 (8.7)	
No. of tumors, n(%)				0.27
Single	137 (55.0)	72 (58.5)	65 (51.6)	
Multiple	112 (45.0)	51 (41.5)	61 (48.4)	
Previous intravesical therapy, n(%)				0.73
None	242 (97.2)	120 (97.6)	122 (96.8)	
BCG	-	-	-	
Doxorubicin	-	-	-	
Mitomycin	7 (2.8)	3 (2.4)	4 (3.2)	
BCG strain, n(%)				0.91

Connaught	5 (2.0)	2 (1.6)	3 (2.4)
Medac	228 (91.6)	113 (91.9)	115 (91.3)
Tice	16 (6.4)	8 (6.5)	8 (6.3)

Supplementary Table 2. Results of the EORTC QLQ-C30 scale at T1 (induction week 6), T5 (maintenance month 6 week 3), and T7 (maintenance month 12 week 3) in a Per Protocol analysis.

	T1 (n=213)			***T5 (n=151)***			***T7 (n=118)***		
**EORTC-Scale**	**SF (n=104)** Mean (SD)	**RF (n=109)** Mean (SD)	**p-value**	**SF (n=75)** Mean (SD)	**RF (n=76)** Mean (SD)	**p-value**	**SF (n=59)** Mean (SD)	**RF (n=59)** Mean (SD)	**p-value**
**Global health status/QoL**									
	71.1 (21.4)	68.4 (19.9)	0.53	70.8 (23.0)	69.1 (22.1)	0.40	70.1 (24.7)	72.8 (22.1)	0.91
**Functional scales**									
Physical functioning	85.0 (19.0)	82.6 (19.5)	0.79	84.8 (18.4)	84.7 (17.5)	0.07	84.1 (18.2)	87.0 (17.0)	0.75
Role functioning	82.2 (25.6)	77.7 (30.2)	0.31	80.2 (27.4)	75.1 (27.7)	0.43	81.6 (25.8)	80.2 (26.5)	0.90
Emotional functioning	82.9 (19.0)	80.1 (23.6)	0.71	81.9 (22.6)	76.1 (25.0)	0.45	82.1 (19.8)	81.3 (21.9)	0.79
Cognitive functioning	89.4 (16.8)	87.3 (18.5)	0.60	87.6 (18.6)	85.5 (20.1)	0.34	87.6 (21.1)	82.8 (22.7)	0.71
Social functioning	86.5 (21.4)	82.7 (22.3)	0.80	83.3 (23.7)	83.6 (21.7)	0.73	85.6 (22.4)	85.6 (21.0)	0.38
**Symptom scales/items**									
Fatigue	24.7 (24.6)	28.9 (26.5)	0.45	27.3 (23.1)	28.9 (25.9)	0.38	23.7 (23.0)	27.5 (26.6)	0.95
Nausea/vomiting	1.3 (5.5)	4.4 (9.8)	0.57	2.5 (7.1)	3.5 (8.7)	0.53	2.8 (7.7)	3.1 (12.2)	0.24
Pain	16.2 (23.6)	20.6 (26.5)	0.56	24.5 (27.9)	18.6 (24.2)	0.27	18.6 (23.8)	26.3 (30.7)	0.62
Dyspnea	17.3 (27.5)	19.3 (26.9)	0.31	16.4 (27.6)	18.2 (27.0)	0.50	16.1 (25.2)	16.9 (25.8)	0.67
Insomnia	21.7 (26.3)	22.6 (30.4)	0.66	21.8 (28.2)	24.9 (29.6)	0.44	21.5 (26.1)	25.4 (29.9)	0.84
Appetite loss	5.8 (15.7)	10.4 (20.1)	0.48	5.3 (13.5)	6.2 (17.1)	0.92	5.6 (16.6)	5.6 (14.1)	0.55
Constipation	7.1 (17.2)	15.3 (26.0)	0.86	8.6 (17.5)	11.4 (22.1)	0.78	10.7 (22.7)	10.7 (22.7)	0.53
Diarrhea	5.8 (15.0)	8.3 (16.5)	**0.01**	4.9 (14.2)	9.8 (21.1)	0.62	6.8 (13.5)	8.0 (16.9)	0.84
**Financial difficulties**									
Financial difficulties	4.8 (16.3)	8.0 (20.3)	0.57	6.2 (18.7)	7.0 (18.3)	0.76	6.2 (15.8)	2.9 (11.3)	0.83

Supplementary Table 3. Incidence of WHO Grade I to IV side effects@ by treatment groups at timepoints T1 (induction week 6), T5 (maintenance month 6 week 3), and T7 (maintenance month 12 week 3) in a Per Protocol analysis.

	T1			***T5***			***T7***		
**Side effect (SE)**	**Standard frequency (n=123)** N (%)	**Reduced frequency (n=126)** N (%)	**p-value**	**Standard frequency (n=123)** N (%)	**Reduced frequency (n=126)** N (%)	**p-value**	**Standard frequency (n=123)** N (%)	**Reduced frequency (n=126)** N (%)	**p-value**
No SE form filled out #	46 (37.4)	49 (38.9)	-	67 (54.5)	64 (50.8)	-	69 (56.1)	67 (53.2)	-
**Local SEs**									
No local SEs reported (grade 0)	38 (30.9)	42 (33.3)	-	25 (20.3)	37 (29.4)	-	16 (13.0)	32 (25.4)	-
Total no. of patients with SEs	39 (31.7)	35 (27.8)	0.52	37 (30.1)	26 (20.6)	0.15	32 (26.0)	22 (17.5)	**<.001**
Frequency	24 (19.5)	22 (17.5)	0.73	26 (21.1)	19 (15.1)	0.37	29 (23.6)	12 (9.5)	**0.003**
Urgency	27 (22.0)	22 (17.5)	0.39	24 (19.5)	18 (14.3)	0.46	26 (21.1)	13 (10.3)	**0.026**
Dysuria	16 (13.0)	10 (7.9)	0.20	13 (10.6)	11 (8.7)	0.86	22 (17.9)	6 (4.8)	**0.001**
Incontinence	8 (6.5)	7 (5.6)	0.79	6 (4.9)	6 (4.8)	0.85	4 (3.3)	6 (4.8)	0.42
Macroscopic hematuria	10 (8.1)	6 (4.8)	0.29	13 (10.6)	11 (8.7)	0.86	11 (8.9)	6 (4.8)	0.26
Bacterial cystitis	2 (1.6)	7 (5.6)	0.09	5 (4.1)	0 (0)	**0.03**	3 (2.4)	2 (1.6)	0.72
Chemical cystitis	3 (2.4)	1 (0.8)	0.31	3 (2.4)	3 (2.4)	0.90	5 (4.1)	0 (0)	**0.03**
Other	3 (2.4)	5 (4.0)	0.47	3 (2.4)	0 (0)	0.10	2 (1.6)	0 (0)	0.17
Total no. of SEs	93	80	0.56*	93	68	0.50*	102	45	**0.004***
**Systemic SEs**									
No systemic SEs reported (grade 0)	61 (49.6)	65 (51.6)	-	40 (32.5)	54 (42.9)	-	44 (35.8)	53 (41.1)	-
Total no. of patients with SEs	16 (13.0)	12 (9.5)	0.40	16 (13.0)	8 (6.3)	0.12	10 (8.1)	6 (4.8)	0.37
Fever	4 (3.3)	8 (6.3)	0.23	8 (6.5)	6 (4.8)	0.71	2 (1.6)	4 (3.2)	0.34
General malaise	11 (8.9)	3 (3.2)	0.06	10 (8.1)	4 (3.2)	0.13	4 (3.3)	2 (1.6)	0.43

Skin rash	0 (0)	2 (1.6)	0.16	0 (0)	0 (0)	-	0 (0)	0 (0)	-
BCG induced lung infection	0 (0)	0 (0)	-	0 (0)	0 (0)	-	0 (0)	0 (0)	-
Sepsis	1 (0.8)	0 (0)	0.32	2 (0.16)	0 (0)	0.18	0 (0)	0 (0)	-
Other	2 (1.6)	2 (1.6)	1.00	2 (1.6)	0 (0)	0.19	4 (3.3)	1 (7.9)	0.12
Total no. of SEs	18	15	0.30*	22	10	0.30*	10	7	0.36*

# For patients for whom a side effect form was not filled out, we assumed there were no side effects.

* Calculated using Mann-Whitney U test based on average number of side effects per patient
